# Killing Me Softly: Connotations to Unfolded Protein Response and Oxidative Stress in Alzheimer's Disease

**DOI:** 10.1155/2016/1805304

**Published:** 2016-01-06

**Authors:** Beata Pająk, Elżbieta Kania, Arkadiusz Orzechowski

**Affiliations:** ^1^Electron Microscopy Platform, Mossakowski Medical Research Centre, Polish Academy of Sciences, Pawińskiego 5, 02-106 Warsaw, Poland; ^2^Department of Physiological Sciences, Faculty of Veterinary Medicine, Warsaw University of Life Sciences (SGGW), Nowoursynowska 159, 02-776 Warsaw, Poland

## Abstract

This review is focused on the possible causes of mitochondrial dysfunction in AD, underlying molecular mechanisms of this malfunction, possible causes and known consequences of APP, A*β*, and hyperphosphorylated tau presence in mitochondria, and the contribution of altered lipid metabolism (nonsterol isoprenoids) to pathological processes leading to increased formation and accumulation of the aforementioned hallmarks of AD. Abnormal protein folding and unfolded protein response seem to be the outcomes of impaired glycosylation due to metabolic disturbances in geranylgeraniol intermediary metabolism. The origin and consecutive fate of APP, A*β*, and tau are emphasized on intracellular trafficking apparently influenced by inaccurate posttranslational modifications. We hypothesize that incorrect intracellular processing of APP determines protein translocation to mitochondria in AD. Similarly, without obvious reasons, the passage of A*β* and tau to mitochondria is observed. APP targeted to mitochondria blocks the activity of protein translocase complex resulting in poor import of proteins central to oxidative phosphorylation. Besides, APP, A*β*, and neurofibrillary tangles of tau directly or indirectly impair mitochondrial biochemistry and bioenergetics, with concomitant generation of oxidative/nitrosative stress. Limited protective mechanisms are inadequate to prevent the free radical-mediated lesions. Finally, neuronal loss is observed in AD-affected brains typically by pathologic apoptosis.

## 1. Introduction

Alzheimer's disease (AD) is known for almost 120 years as a progressive fatal human neurodegenerative disease featured by decline in both memory and cognitive functions [1]. Early-onset familial AD (FAD) which accounts for less than 5% of cases is linked to mutations in* APP* gene on chromosome 21 or genes encoding components of *γ*-secretase (presenilin 1, presenilin 2) resulting in increased A*β*_42/40_ ratio, where A*β*_42_ is highly fibrillogenic [2–4]. Sporadic, or late-onset, AD (SAD) which is a major form (over 95% of cases) has unknown etiology. While one out of nine people aged 65 or older has Alzheimer's, nearly one out of three people aged 85 or older has the disease [5]. The underlying molecular mechanisms that cause the formation of the hallmarks of FAD and SAD, namely, amyloid-*β*- (A*β*-) containing plaques and microtubule-associated protein tau-containing neurofibrillary tangles (NFTs), are not yet fully clarified. The A*β* peptide is a cleavage product of amyloid precursor protein (APP) by sequential action of *β*- and *γ*-secretases which release 39–43-amino-acid peptide from the C-terminal (cytoplasmic) end of transmembrane protein [6]. The outcome of the end-stage intracellular lesions in FAD and SAD is loss of neurons (brain atrophy) with most affected regions as frontal cortex, hippocampus, and amygdala [7]. Severe injuries are very selective and restricted to neurons as shown by the morphometric analyses of brain tissue slices obtained at autopsy from cases with diagnosis of AD faced up with cases with no clinical or pathological history of neurological disease [8]. Bulk of changes is characterized by significant reduction in mitochondria density, accumulation of mtDNA and proteins in cytoplasm and in the vacuoles associated with lipofuscin once involved in mitophagy [9]. These observations show increased mitochondrial degradation products either by autophagy or by messed up proteolytic systems. Mitochondrial abnormalities to milder extent were also found in other cell types (endothelium, fibroblasts) obtained from patients with AD [10, 11]. We also observed extensive autophagy in cellular model of FAD and SAD [12].

For decades, the hypothesis of AD (“amyloid cascade hypothesis”) of extracellular amyloid-*β* plaques and intracellular NFTs accumulations as clues in AD pathogenesis have been extensively examined with conflicting results. In the last decade, however, a new attractive hypothesis emerged from studies concerning mitochondria as key organelles for maintaining neuron functions and survival. A growing body of evidence supports the idea that dysfunctional mitochondria cause development of synaptic abnormalities, neuronal degeneration, and ultimately cell death as a consequence of unbearable oxidative stress in AD [13–16]. Numerous* in vitro* and* in vivo* experiments substantiated the so-called “vicious cycle hypothesis,” pointing to the importance of mitochondria in the pathogenesis of AD [17–28]. Due to their limited glycolytic capacity (lack of salvage pathway), neurons are highly dependent on mitochondrial function for energy release and severely affected by the limited oxygen and glucose supply, making them especially susceptible to energy dyshomeostasis [29]. Moreover, mitochondria, which produce almost entire energy in neurons, have recently been found to be targeted by APP and A*β* [15, 17, 30–35]. The presence of APP and A*β* in mitochondria has detrimental consequences as both constituents cause perturbations in cellular energy homeostasis.

## 2. Neurons: Cells Extremely Susceptible to Energy Dyshomeostasis

Excitability is a basic attribute of neurons (as well as other excitable cells), as it encompasses the primary task to receive, analyze, and dispatch electronic signals within the neuronal network or to their cognate effectors. This function is achieved by the generation of electric currents, some of which are of high frequencies. These electric currents are evoked by the ion fluxes (Na^+^, K^+^, Ca^2+^, and Cl^−^) through channels located in the plasma membrane. Any change in the concentration of K^+^ or Na^+^ at the extra- or intracellular site of plasma membrane, respectively, activates Na^+^/K^+^-ATPase which restores the concentration gradient essential for excitability and also controls the cell volume. The active transport against the concentration gradient is entirely dependent on ATP delivered to Na^+^/K^+^-ATPase and other pumps (Ca^2+^-ATPase, H^+^-ATPase). ATP is hydrolyzed leading to phosphorylation of the pump at a highly conserved aspartate residue and subsequent release of ADP. Energy generation and energy consumption are tightly coupled to neuronal activity at the cellular level. Na^+^/K^+^-ATPase, a major energy-consuming enzyme, is well expressed in neurons rich in cytochrome *c* oxidase, an important enzyme of the energy-generating machinery and glutamatergic receptors that are mediators of neuronal activity [36]. Na^+^/K^+^-ATPase enzyme consumes the bulk of energy in the brain [37–39]. Nervous cells are highly enriched in mitochondria, the main energy supplying organelle, which provide ATP once they are sufficiently supplied with oxygen. Mitochondrial ATP is exchanged with cytosolic ADP through inner membrane adenine nucleotide translocase, so the intracellular location of mitochondria is crucial for availability of ATP and accelerated by cytosolic ADP. To meet energy requirements, mitochondria move regularly along the microtubular meshwork to the sites of higher ATP demand (high concentration of ADP), where they undergo the fusion process. Mitochondrial ATP is also indispensable energy donor for dynamins (kinesin, dynein), the proteins responsible for microtubule-associated axonal transport of the secretory vesicles. Obviously, any substantial interruption of the mitochondrial function, distribution, and fusion would affect the ATP delivery with resultant defects in neuronal activity.

## 3. APP Processing and A*β* Formation

**Figure 1:**
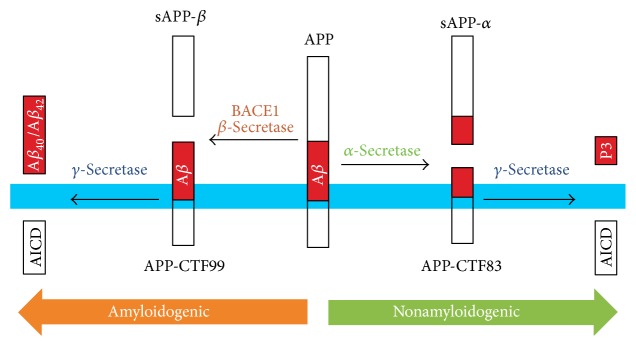
A diagram of amyloid precursor protein (APP) processing pathway. The transmembrane protein APP (membrane indicated in blue) can be processed by two pathways: the nonamyloidogenic *α*-secretase pathway and the amyloidogenic *β*-secretase pathway. In the nonamyloidogenic pathway, *α*-secretase cleaves in the middle of the *β*-amyloid (A*β*) region (red) to release the soluble APP-fragment sAPP-*α*. The APP C-terminal fragment 83 (APP-CTF83, *α*CTF) is then cleaved by *γ*-secretase to release the APP intracellular domain (AICD) and P3 fragment. In the amyloidogenic pathway, *β*-secretase cleaves APP to produce the soluble fragment sAPP-*β*. APP-CTF99 (*β*CTF) is then cleaved by *γ*-secretase to produce A*β*_40_, A*β*_42_, and AICD. Adopted from [179].

First, widely expressed APP is a transmembrane glycoprotein, synthesized on three different templates (APP695, APP751, and APP770) which resulted from alternative splicing of the transcript [40, 41]. Second, after APP is synthesized on polysomes, this protein undergoes N-glycosylation in the ER. Once it is N-glycosylated, the APP is then transported to the Golgi apparatus. Golgi apparatus is a second chief site of APP posttranslational modifications including O- and N-glycosylations, phosphorylations, and sulphonations [42, 43]. Great deal of mature APP protein is stored in Golgi and trans-Golgi network (TGN), while approximately 10% of APP is unidirectionally (anterograde) transported by kinesins in TGN vesicles or in elongated tubular structures along microtubules in soma, dendrites, and axons [44, 45]. Third, APP glycoprotein embedded to plasma membrane is preferentially cleaved in the nonamyloidogenic pathway; alternatively it could be internalized via endocytosis [46]. Endosomic APP protein as well as its processed fragments can return to plasma membrane, can be proteolytically degraded in the lysosome, or can be transported from early endosome to TGN. Retention of APP in the endoplasmic reticulum/intermediate compartment (ER/IC) eliminated production of intracellular A*β*_40_ but did not alter synthesis of fibrillogenic form (A*β*_42_) [47]. Interestingly, the production of intracellular A*β* from wild-type APP695 appears to be a unique characteristic of postmitotic neurons, since intracellular A*β* was not detected in several nonneuronal cell lines [48]. Whether APP retromer (transported from early endosome to TGN) is also cleaved via amyloidogenic pathway is not clear due to conflicting observations [49, 50]. In neurons, APP695 is the major isoform and could be subject to sequential proteolytic cleavage by *β*- and *γ*-secretase to free A*β*. The *β*-secretase (BACE1, transmembrane aspartyl protease) initiates endoproteolytic cleavage giving rise to N-terminus of A*β* (*β*-secretase cleaved APP to *β*CTF as the intermediate) followed by *γ*-secretase (membrane-embedded aspartyl protease complex consisting of presenilin, PS), presenilin enhancer-2 (Pen-2), anterior pharynx defective-1 (Aph-1), and nicastrin, which reveals the C-terminus of A*β* [51]. Given that two PS (PS1 and PS2) and Aph-1 (Aph-1A and Aph-1B) variants exist, the processing of APP by four different human *γ*-secretase complexes each acting at more than one *β*CTF site (*ε*-, *ζ*-, and *γ*-) leads to formation of several A*β* (A*β*_37–43_), with A*β*_40_ and A*β*_42_ being predominant species. Finally, three end products are formed (sAPP-*β*, A*β*, and amyloid precursor protein intracellular domain, AICD). The key neuron *α*-secretase (ADAM10) cleaves APP inside A*β* polypeptide chain to *α*CTF as the intermediate, so after subsequent *γ*-secretase action on *α*CTF, three nonpathogenic fragments are formed (sAPP-*α*, P3 fragment, and AICD) (Figure 1).

As aforementioned, FAD is caused by mutations in* APP* and* PSEN* genes located on chromosomes 21 and 14, respectively, but the incidence of AD is also higher in dominantly inherited duplications of the APP locus in elderly individuals with Down's syndrome (trisomy of the 21st chromosome), pointing to important role played by APP and A*β* in AD. Mutations in APP located near the *β*-secretase cleavage site increase production of A*β*, whereas those near the *γ*-secretase cleavage site result in an increased ratio of A*β*_42_ to A*β*_40_ [52]. The *ε*4 allele of apolipoprotein E is the major risk factor for SAD. Thus, this particular* APOE* gene polymorphism increases disease risk in a dose-dependent manner and lowers the age of onset, as shown by Corder et al. [53]. One copy of APOE4 increases the risk of AD about fourfold (compared with the more common APOE*ε*3/APOE3*ε*3 genotype), whereas two copies of APOE4 increase the risk of AD about 12-fold. The mechanism by which the amino acid difference between APOE3 and APOE4 increases the risk of AD remains to be established.

Widespread occurrence of APP and A*β* in nervous system brought about the assumption that both components might play physiological roles. More than few possible concepts have emerged, some validated by experimental data. The APP protein overexpression led to enhanced survival and growth of some cell types [54, 55]. Furthermore, secreted forms of APP (APP^s^s: sAPP*α* and sAPP*β*) were antiapoptotic [56] and have a potent neuroprotective action in cultured rat hippocampal and septal neurons and in human cortical neurons [57]. APP^s^_695_ and APP^s^_751_ protected neurons against hypoglycemic damage, and the neuroprotection was abolished by antibodies to a specific region common to both APP^s^_695_ and APP^s^_751_. Thus, APP^s^s may normally play excitoprotective and neuromodulatory roles. Accordingly, APP was shown to stimulate axon branching and the maintenance and formation of synapses, neuronal survival, and neuritic outgrowth [58–60]. APP protein is highly expressed in axons and interacts with extracellular matrix components [61–64]. Similar to APP, A*β* was demonstrated to play a physiological role in synaptic plasticity as minute quantities of the peptide stimulated neurons and enhanced the release of neurotransmitter [65, 66]. Everything can change when the things go awry.

## 4. Perturbations in ER

**Figure 2:**
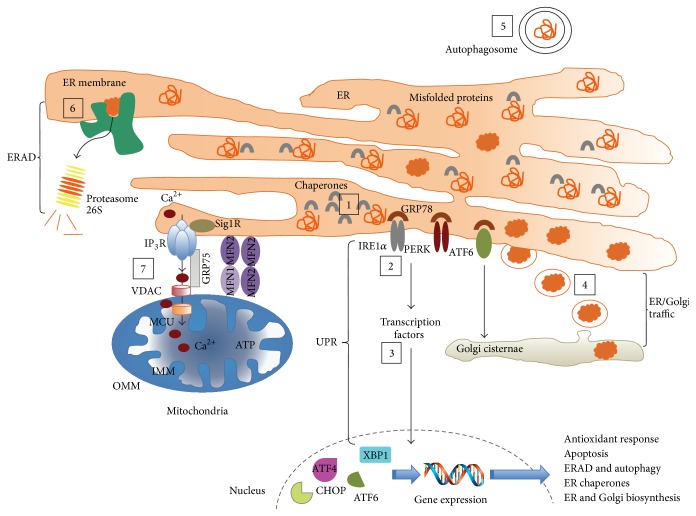
ER stress triggered by misfolded proteins in several neurodegenerative diseases. Abnormal conformations of the proteins APP, A*β*, and tau are implicated in the pathogenesis of AD. Alterations in the function of ER chaperones and UPR-related components, ERAD, ER/Golgi trafficking, and ER-to-mitochondria Ca^2+^ transfer have been suggested as underlying mechanisms of ER stress triggered by these disease-associated proteins. These proteins can accumulate and aggregate at the ER and their stable interaction with ER chaperones such as GRP78/BiP and PDI may trap ER chaperones, altering protein folding with concomitant ER stress. In addition, these proteins can lead to the oxidative modification of the active site of PDIs by nitrosylation leading to their enzymatic inactivation. Furthermore, some of these proteins alter the activity of the UPR stress sensors (IRE1*α*, PERK, and ATF6) as well as the activity/levels of downstream signaling mediators and transcription factors, including cleaved ATF6, ATF4, and spliced XBP1. As a result, genes implicated in autophagy and ERAD, antioxidant response, ER chaperones, and organelle's biosynthesis are upregulated. Moreover, these proteins block the exit of vesicles from the ER and alter the trafficking between ER and Golgi of properly folded proteins. The cellular responses controlled by UPR transcription factors, including the modulation of autophagy mediated degradation of protein aggregates, become compromised. Disease-related proteins can also interact with ERAD components, precluding the translocation of ERAD substrates from the ER to the cytosol, leading to the accumulation of abnormally folded proteins at the ER. Finally, Ca^2+^ released from the ER, mainly through the IP_3_R, and its transfer to mitochondria can be impaired in the presence of disease-related proteins leading to mitochondrial Ca^2+^ overload and activation of apoptotic cell death pathways. AD: Alzheimer's disease; ATF6: activating transcription factor 6; ATF4: activating transcription factor 4; ER: endoplasmic reticulum; ERAD: endoplasmic-reticulum-associated protein degradation; IP_3_R: inositol triphosphate receptor; IRE1*α*: inositol-requiring enzyme 1 alpha; PERK: protein kinase R- (PKR-) like ER kinase; UPR: unfolded protein response; XBP1: Xbox binding protein 1. Adopted from [67].

In healthy cells including neurons, ER is a fundamental organelle for protein quality control in the secretory pathway, which prevents protein aberrant folding and aggregation [67]. A bulk of evidence shows the importance of ER in APP maturation and processing. With regard to APP intracellular processing, both secretases (*α*- and *β*-) have been identified in the ER together with *γ*-secretase which is present in mitochondria-associated membrane (MAM) subcompartment [48, 68]. This distinctive intracellular lipid-raft-like structure is involved in cholesterol and phospholipid metabolism, Ca^2+^ metabolism, and mitochondrial dynamics and becomes markedly augmented in AD [69]. MAM is responsible for the communication between the ER and the mitochondria with efficient transfer of Ca^2+^ from the ER to mitochondria supporting metabolic functions and cell viability [70]. The molecular bridges between ER inositol 1,4,5-triphosphate receptor (IP_3_R) and the voltage-dependent anion channels in the outer mitochondrial membrane are brought together through the cytosolic chaperone glucose-regulated protein 75 (GRP75) (Figure 2). Additionally, the dynamin-related GTPase mitofusin 2 (Mfn2) proteins located on the ER intermingle with Mfn1 or Mfn2 on mitochondria to tighten the connection. The distance between ER and mitochondria controlled by the phosphofurin acidic cluster sorting protein 2 (PACS-2) of ER and the dynamin-related GTPase protein 1 (Drp1) is crucial for cell survival, as either too long (lack of Ca^2+^ flux) or too short distance (Ca^2+^ overload) might lead to apoptosis [71]. Alternatively, impaired mitochondrial bioenergetics with reduced cellular ATP levels stimulate autophagy. The molecular mechanism of ER-mediated autophagy is accurately regulated by Beclin 1 as well as ER membrane bound protein Bax inhibitor 1 (BI-1). Both proteins are capable of promoting autophagy through IP_3_R-dependent mechanism [72].

## 5. APP Processing and ER Stress Response

The nature of APP processing is determined by the composition of membrane, with cholesterol rich lipid rafts as the site of amyloidogenic cleavage [73]. Consequently, one might expect that fate of APP is at least partly established by the representation of lipid rafts and possible access to *β*- versus *α*-secretase. Irrespective of the type of cleavage, there is one underestimated biochemical step in ER that might bring about damaging upshot. It is apparently the effectiveness of ER situated N-glycosylation which makes the APP molecule suitably folded. If misfolded/malfolded protein(s) accumulate in the ER, complex cascade of reactions known as the unfolded protein response (UPR) is triggered with the so-called endoplasmic reticulum stress response (ERS). Today, it is widely accepted that, during UPR, the ER sensors, protein kinase R- (PKR-) like ER kinase (PERK), activating protein kinase 6 (ATF6), and inositol-requiring enzyme 1 alpha (IRE1*α*), are freed from GRP78/BiP protein repression, which hereafter become activated. Without going into details of individual sensor action, a number of reactions occur at both the genomic and cytoplasmic level with selective degradation of mRNAs encoding protein(s) with abnormal folding and inhibition of protein translation, except for genes important for UPR, redox homeostasis, energy metabolism, and protein folding [67]. On the one hand, PERK phosphorylates eukaryotic initiation factor 2 alpha (eIF2*α*) to stop entrance of methionyl-tRNA to the ribosome; on the other hand, it allows translation of activating transcription factor 4 (ATF4) gene. IRE1*α* sets off alternative splicing of Xbox binding protein 1 (XBP1) transcript leading to activation of the transcription factor liable to stimulate ER/Golgi biogenesis and formation of proteins involved in endoplasmic-reticulum-associated protein degradation known as ERAD (Erdj4, p58^IPK^, EDEM, RAMP-4, PDI-P5, and HEDJ; for details, see [67]). Finally, ATF6 is activated in the Golgi complex through proteolytic cleavage and translocates to nucleus where it cooperates with XBP1 in upregulation of chaperones and ERAD-related genes [43]. In principle, UPR is activated to restore ER homeostasis and stop the accumulation of aberrantly formed protein(s) but if the strength of ER stress is unbearable (meaning that it cannot be compensated by UPR) there is a path to activate apoptosis. Among other routes the most central role is played by the major proapoptotic transcription factor C/EBP homologous protein CHOP/growth arrest and DNA damage induced gene 153 GADD153. It downregulates the antiapoptotic protein Bcl-2 and upregulates proapoptotic Bax and Bak [74]. CHOP/GADD153 leads to excessive production of reactive oxygen species (ROS) within ER, subsequent depletion of reduced glutathione (GSH) and Ca^2+^ flux from the ER to cytoplasm through the IP_3_R [75]. One hitherto unresolved issue is the physiologic importance of APP and GRP78/BiP interaction disclosed in coprecipitation study carried out by Yamamoto et al. [76]. Bulk of APP associated with GRP78/BiP was immature protein. Given that GRP78/BiP expression levels declined in samples of brain tissue obtained from FAD patients as demonstrated by Katayama et al. [77], few distinct scenarios are possible. First, binding to GRP78/BiP suggests ER accumulation of immature APP. Second, APP interaction with GRP78/BiP is a noticeable sign of UPR which is further validated by downregulation of GRP78/BiP in FAD patients. Finally, retention of APP in ER is most likely a result of amyloidogenic APP processing and as such it probably occurs in MAM, the cholesterol enriched domains. ER-mitochondria crossing point is therefore of particular interest in deciphering the links between the ER placed APP processing, UPR, and resulting cellular responses such as autophagy, apoptosis, and inflammatory reaction observed in AD (Figure 2).

## 6. Pathology of Tau Protein

Microtubule-associated protein tau controls assembly and prevents microtubules from severing. Microtubular network is fundamental component of cytoskeleton essential for intracellular transport of secretory vesicles and organelles (i.e., mitochondria). Glycogen synthase kinase 3 beta- (GSK-3*β*-) targeted hyperphosphorylation of tau causes this protein to dissociate from microtubules. Consequently, microtubules become fragmented and microtubule-dependent transport system fails. Furthermore, hyperphosphorylated tau (P-tau) is prone to form oligomers and toxic filaments, known as NFTs or tauopathy [78]. A variety of tau conformers were reported to exist, pointing to different tauopathies capable of self-propagation [79, 80]. Interestingly, intracellular tau inclusions define AD as clinical symptoms of disease observed when tauopathy is abundant together with intracellular A*β* deposits in neocortex [51, 81, 82]. People with abundant A*β* plaques, but no or only a few neurofibrillary lesions, do not have AD. Clinicopathological correlation studies have been crucial to generate hypotheses about the pathophysiology of the disease, by establishing the fact that there is a continuum between “normal” aging and AD dementia and that the amyloid plaque buildup occurs primarily before the onset of cognitive deficits, while neurofibrillary tangles, neuron loss, and particularly synaptic loss parallel the progression of cognitive decline [83]. Thus, misfolded proteins and descendant toxic filaments with a number of intermediates are critical for manifestation of AD, as fibrillogenic APP processing is not enough for onset of disease. Although the molecular mechanism of tauopathy is not deciphered in full, recent reports suggest ER stress as the starting point [84, 85]. This idea is substantiated by the elevated levels of ERS and UPR markers together with P-tau and GSK-3*β* in brains affected by AD [86]. Therefore, on the one hand, the incidence of UPR is strongly correlated with the presence of NFTs; on the other hand, aggregation of P-tau induces ERS with resultant UPR. Some lines of evidence confirmed UPR activation near the beginning of NFTs formation and point to the functional link between malformed tau protein and UPR. The* in vitro* experiments with phosphatase 2A inhibitor or phosphorylation activator demonstrated enhanced P-tau formation in neurons in concert with the increased levels of PERK, eIF2*α*, and XBP1 transcript, apparent markers of UPR [87]. Furthermore, GRP78/BiP was found to encourage tau phosphorylation through facilitated substrate capture by GSK-3*β* [88]. GSK-3*β* seems to play dual role; first this kinase protects neurons from apoptosis as P-tau accumulation is strong molecular signal to trigger UPR with subsequent autophagy. Second, UPR raises GSK-3*β* activity through lysosomal degradation of inactive GSK-3*β* (P-Ser9-GSK-3*β*). To sum up, ERS and UPR are important molecular machines used to prevent cell viability turned on by tauopathy.

## 7. Oxidative Stress in ER

Sacs and tubes of ER delineate the compartment where the newly synthesized proteins undergo maturation to native state. Native state indicates properly folded, fully functional protein. Important reactions essential for protein folding of unbranched polypeptide chains include amino acids oxidation and glycosylation. As a result, redox homeostasis in ER is shifted to oxidative state so as to promote disulfide bond formation between adjacent cysteines. Oxidation of sulfhydryl groups required to make disulfide bonds is controlled by ER oxidase 1*α* (ERO1*α*). Next, disulfide bonds could be subject to posttranslational modification, disulfide exchange by protein disulfide isomerase (PDI). PDI is able to correct mispaired thiol residues by catalyzing the breakage and formation of correct disulfide bonds. These enzymes are fundamental for protein folding. Oxidation allows twisting of proteins, which is followed by N-glycosylation and/or C- and O-mannosylation. Protein N-glycosylation in eukaryotes is a complex process divided into several steps. First, there is a “call for” carrier lipids (polyisoprenyl phosphates such as dolichyl phosphates), the membrane lipids known to function as glycosyl transporters. In mammalian cells, the limiting substrate for dolichol biosynthesis is geranylgeraniol (GGOH) of mevalonate pathway. Dolichols, the longest aliphatic molecules synthesized in animal cells, have 18–21 *α*-isoprene saturated units (C90–105), critical for their recognition by the enzymes (glycosyltransferases) that glucosylate dolichyl phosphates [89]. Once dolichyl monophosphates (Dol-P) are formed in the ER membrane, the precursor oligosaccharide donor (GLc_3_Man_9_GLCNAc_2_-P-P-dolichol) for protein N-glycosylation can be synthesized on the lumenal leaflet of ER. First, three sugar intermediates are produced (Man-P-Dol, Glc-P-Dol, GlcNAc-P-P-Dol, and Man_5_GlcNAc-P-P-Dol) on the cytoplasmic leaflet of the ER. Next, enzyme flippases mediate transbilayer movement of the aforementioned intermediates to lumenal side of ER where conversion to Glc_3_Man_9_GlcNAc_2_-P-P-Dol could be completed. Glc_3_Man_9_GlcNAc_2_-P-P-Dol is also used for biosynthesis of glycosylphosphatidylinositol (GPI) anchors.

Taken together, lipid-mediated glycosylation plays a vital role in the appropriate protein folding and intracellular translocation of N-linked glycoproteins [90]. Likewise, it is important for protein O- and C-mannosylation, and GPI anchorage. Moreover, Dol-P availability in the ER is the rate-limiting factor in the production of glycolipid intermediates and N-glycosylation.

## 8. ER Stress and Apoptosis

It was shown that during ERS the ER resident proapoptotic cysteine protease known as caspase (caspase-12 in rat, caspase-4 in humans) is activated through cleavage. As a result caspase cascade is started via caspase-9 that in turn stimulates effector caspase-3 [91, 92]. The central role played by ER in programmed cell death is achieved by PERK branch where ATF4 induces the expression of CHOP/GADD153, which represses antiapoptotic Bcl-2 family proteins and simultaneously shuffles ER membrane Bax and Bak proteins into outer mitochondrial membrane. Consequently, pores are formed to leak the components of apoptosome from mitochondrial intermembrane space [93]. Another important mechanism of ERS-induced apoptosis is led by Ca^2+^-dependent ERO1*α*-IP_3_R pathway where ERO1*α* collaborates with IP_3_R in Ca^2+^ efflux from ER to mitochondria via MAM [75, 94]. Accordingly, Ca^2+^ influx facilitates cytochrome *c* release from mitochondria; besides, cytochrome *c* can bind to ER IP_3_R and the complex amplifies the apoptotic signal in a feedforward manner [95]. Last but not least, ERS-associated apoptotic programme is set off by c-Jun N-terminal kinase (JNK) as the effect of IRE1*α* complexed with TNF-receptor-associated factor 2 (TRAF2) activation of apoptosis-signal-regulating kinase 1 (ASK1) [96, 97].

What does ERS drive to induce apoptotic death in neurons? Actually, many reports indicate that APP and A*β* as well as hyperphosphorylated tau have been shown to block mitochondrial transport, which results in impaired energy storage and oxidative stress [98–101]. Indeed, accumulation of APP, A*β*, and NFTs in mitochondria led to reduced activity of some enzymes involved in substrate oxidation (tricarboxylic acid cycle), electron transport chain (ETC), and ATP synthase, as well as severely diminishing import of nuclear-encoded proteins [26, 27, 102–104]. One may ask if there is any additional link between ER and mitochondria other than MAM which could account for apoptotic signal. Though not directly, ER significantly contributes to oxidative stress in mitochondria of AD-affected subjects.

## 9. Abnormal APP Processing and Trafficking Culminate in ER Pathology of AD

From the morphological point of view, as neurons are highly specialized cells, soma, dendrites, and neurites are considerably distinct structures. Proteins needed by these compartments are delivered via microtubules once proteins have suitable sorting signals (i.e., APP trafficking from ER to plasma membrane is associated with several posttranslational modifications with oxidation and N-glycosylation). Additionally, the Golgi apparatus follows ER in subsequent APP adjustment (O- and N-glycosylation, phosphorylation, and sulphonation) [42]. Any inaccurate alteration of the APP molecule is potentially hazardous, as protein final destiny is missed causing its retention in ER or trans-Golgi network (TGN). In addition, other unusual settings for APP are possible as this large protein has few signal sequences hidden when APP is correctly folded. In the cells transfected with APP, this protein enters coat protein complex I (COPI) vesicles and undergoes retrograde transport from cis end of the Golgi complex back to the ER [76]. Such response causes accumulation of APP in the tubulocisternal ER system together with aberrant intracellular translocation of the protein. Interestingly, the issue whether APP is subject to retrograde transport with successive fibrillogenic processing because of UPR and its interaction with GRP78/BiP is not clear, as the observations are inconsistent [77]. Nonetheless, accumulation of misfolded/malfolded proteins in the ER suggests disorganized process of posttranslational change. As anticipated, the accretion of proteins of anomalous pattern signals ERS and UPR followed by increased vulnerability to apoptotic cell death. Prior to decay, however, APP protein levels in the ER lessen by dint of cleavage with *β*- and *γ*-secretase [105, 106]. Products of this cleavage (sAPP*β*, A*β*, and AICD fragment) all appreciably influence neuronal survival most likely through nonnative form of the APP substrate.

## 10. APP, A*β*, and NFTs Mark Mitochondria as Targeted in AD

Mitochondrial import of A*β*_40_ and A*β*_42_ peptides through the translocase TOM complex was blocked by preincubation of isolated mitochondria with antibodies raised against TOM proteins (TOM20, TOM40, and TOM70) [107]. Neither VDAC inhibition with antagonist antibodies nor inhibition of mitochondrial permeability transition pores (MPTP) or fall of mitochondrial membrane potential (MMP) affected uptake of A*β* [17]. With regard to APP, elegant study performed by Anandatheerthavarada and his colleagues [30] revealed that C-terminal truncated APP (lacking A*β*) targets mitochondria in cholinergic, GABAergic, dopaminergic, and glutamatergic neurons of AD brain by clogging up mitochondrial protein translocase complex TOM40/TIM22. Authors propose that occlusion of translocase is followed by blunted import of nuclear-encoded proteins vitally important for energy homeostasis. Mitochondrial APP protein transmembrane orientation indicates NH_2_-terminal inside in contact with translocase, whereas COOH-terminal is facing cytoplasmic side. NH_2_-terminus has mitochondrial signal sequence. Astonishingly, mitochondrial APP molecules were nonglycosylated giving rise to speculation that protein molecules that arrived at mitochondria have not achieved molecular maturity [31]. The accumulation of nonglycosylated APP species in mitochondrial import channels of AD brain was directly related to decreased mitochondrial functions as validated by the decline in cytochrome *c* oxidase activity (complex IV) and elevated levels of H_2_O_2_. Furthermore, in AD brain, the mitochondrial accumulation of nonglycosylated APP went along with a corresponding reduction in plasma membrane-associated APP. It suggests that AD brain has APP processing and trafficking severely affected by incomplete N-glycosylation, ensuing ER protein accumulation and exposure of the cryptic mitochondrial targeting signal for assisting chaperone proteins. Several lines of evidence indicate that not fully formed proteins are phosphorylated and bind to the cytosolic proteins required for movement from ER to mitochondria [108–110]. Moreover, ERS and UPR in AD seem to be incompetent and inefficient in elimination of malfolded proteins. In addition to APP also A*β* was frequently reported to occupy mitochondria although its origin and mechanism of mitochondria targeting mostly remain unexplored (except for involvement of protein translocase TOM complex). The possibility of mitochondrial A*β* generation has to be ruled out, as membrane orientation of arrested APP does not allow access to *γ*-secretase (*γ*-secretase activity in mitochondria was detected by independent study) [111]. Probably, A*β* species is derived from APP prior to its translocation (ER?), so A*β* may be transported to mitochondria independently of APP. Collectively, observations showing mitochondrial presence of APP, A*β*, and tau in aberrant configuration point toward the anomaly of protein folding at the level of ER and Golgi apparatus. Nonglycosylated molecule of APP suggests defective transfer of sugar core from dolichyl phosphate(s) and further modifications such as O- and N-glycosylations. One may admit that the lack of glycosyl residue brings about pathologic processing and trafficking of APP and its fragments. In point of fact, dolichol derivatives, mixture of polyprenols (acyclic isoprenoid alcohols) known as Ropren (Solagran Limited, Melbourne, Australia) commercially used to treat liver diseases, were tested in the treatment of AD in two separate trials conducted in 2005 and 2006 with promising results [http://www.asx.com.au/asxpdf/20071119/pdf/315x5nh4hm8wv7.pdf, http://www.asx.com.au/asxpdf/20070221/pdf/3111ztwcbqzkk9.pdf]. Further studies are urgently needed to test how important the glycosylation process is in the pathogenesis of AD. Dolichols are obtained from geranylgeraniol (GGOH) and the latter is an intermediate of mevalonate pathway. GGOH is a common substrate for dolichol and ubiquinone synthesis, but it is also necessary for protein prenylation. As both GGOH and farnesol (FOH) are engaged in protein prenylation more concern should be laid on the importance of these compounds in AD pathogenesis. Observations demonstrating mitochondrial relocalization of other proteins without posttranslational modification seemingly point to increased mitochondrial targeting of immature molecules resulting in mitochondrial dysfunction and acceleration of disease progression [34, 112–114].

## 11. Oxidative Stress in Mitochondria of AD Brains

**Figure 3:**
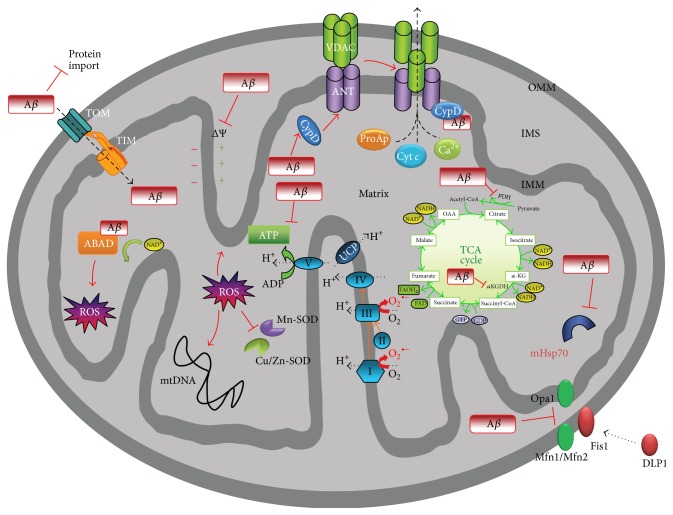
Amyloid-*β*-related mitochondrial impairment. Mitochondria were found to be the target for amyloid-*β* (A*β*), which interacts with several proteins, leading to mitochondrial dysfunction. Indeed, A*β* was found in the outer mitochondrial membrane (OMM) and inner mitochondrial membrane (IMM) as well as in the matrix. The interaction of A*β* with the OMM affects the transport of nuclear-encoded mitochondrial proteins, such as subunits of the electron transport chain complex IV, into the organelle via the translocase of the outer membrane (TOM) import machinery. Moreover, A*β* disturbs the activity of several enzymes, such as pyruvate dehydrogenase (PDH) and oxoglutarate dehydrogenase (OGDH), decreasing NADH reduction, and the electron transport chain enzyme complex IV, reducing the amount of hydrogen that is translocated from the matrix to the intermembrane space (IMS), thus impairing the mitochondrial membrane potential (MMP). Taken together, these events cause abnormal mitochondrial electron activities, leading to decreased complex V activity and so to a drop in ATP levels, in addition to increasing reactive oxygen species (ROS) generation. Moreover, ROS induce peroxidation of several mitochondrial macromolecules, such as mitochondrial DNA (mtDNA) and mitochondrial lipids, contributing to mitochondrial impairment in the mitochondrial matrix. The complex of A*β* bound to binding alcohol dehydrogenase (ABAD) impairs the binding of NAD^+^ to ABAD, changes mitochondrial membrane permeability, and reduces activities of respiratory enzymes, inducing further ROS production and leading to mitochondrial failure. A*β* binding also activates Fis1 (fission protein) and promotes increased mitochondrial fragmentation; this increased mitochondrial fragmentation produces defective mitochondria that ultimately damage neurons. Furthermore, A*β* binding to cyclophilin D (CypD) enhances the protein translocation to the inner membrane, favouring the opening of the mitochondrial permeability transition pore, formed by the adenine nucleotide translocator (ANT) and voltage-dependent anion channels (VDACs). Cyt *c*: cytochrome *c*; DLP1: dynamin-like protein 1; PDH: pyruvate dehydrogenase; ProAp: proapoptotic factors; SOD: superoxide dismutase; TCA: tricarboxylic acid; TIM: translocase of the inner membrane. Adopted from [14].

Normal physiological functions of APP are thought to be involved in the stabilizing contact points between synapses and maintaining mitochondrial functions [60, 115]. Mitochondrial dysfunction was often observed regardless of the experimental model used to study AD [8, 29, 116–119]. It includes defects in oxidative phosphorylation, decreased ATP, decreased membrane potential, increased production of ROS/RNS, and perturbation in mitochondrial fusion and fission [15, 30, 31, 115, 120–122]. Hyperphosphorylated tau was also reported to impair mitochondrial functions [123]. Using proteomic approach, the strongest defects of the respiratory capacity were observed mainly at complexes I, IV, and ATP synthase (complex V) at both protein and activity level [124]. While APP, A*β*, and hyperphosphorylated tau are potent inhibitors of mitochondrial import of nuclear-encoded proteins, apparently the pathology of each leads to metabolic harm in different way. In freshly isolated mitochondria from AD brains, the APP inhibited mitochondrial import of cytochrome *c* oxidase (COX) subunits IV and Vb [31]. Dysfunction of COX increases ROS production (incomplete reduction of oxygen molecules), reduces energy stores, and disturbs energy metabolism. Accordingly, in AD patients, deficiency of COX was found in brains and platelets [119, 125, 126]. Similar to APP, A*β* was found in mitochondria of transgenic mice and cellular and human AD models [17, 19, 33, 103, 127–130]. At present it is not clear whether the observed mitochondrial toxicity is due to APP or A*β*, or NFTs accumulation. Anyway, some regularity is observed with respect to most affected components of respiratory chain. NADH-ubiquinone oxidoreductase (complex I) activity is reduced to the utmost by hyperphosphorylated tau, whereas decreased activity of cytochrome *c* oxidase (complex IV) that resulted in mitochondrial dysfunction was observed during A*β* and APP accumulation [14, 15, 31, 32, 34]. Concomitantly, rise in the activity of antioxidant enzymes manganese superoxide dismutase (Mn-SOD) and catalase (CAT) was demonstrated in response to elevated levels of free radicals including superoxide anion radical (O_2_^∙−^), hydroperoxyl radical (HO_2_^∙^), hydroxyl radical (OH^∙^), and nitric oxide radical (NO^∙^) [15]. Consistent with observations of chronic respiratory chain dysfunction and mitochondrial oxidative stress, there are reports showing their contribution to tau pathology in AD [131]. In any case, free radicals that override antioxidant defense react with a wide variety of organic components causing lipid peroxidation to advanced lipid oxidation end products (ALE), cross-linking of proteins, nitrosylation of proteins, and mutations in DNA. Mitochondrial circular DNA (mtDNA) of ~16 kbp is devoid of repair systems meaning the buildup of lesions. There are 37 genes located in mtDNA with those encoding protein subunits of complex I (7), complex II (1), and complex IV (3) but not complex III. Interestingly, hallmarks of AD (APP, A*β*, and NFTs) mostly affect members of electron transfer chain (complexes I and IV) which rely exclusively on mitochondrially predetermined subunits. Maybe it is not simple coincidence, but the effect of inhibited import to mitochondria of nuclear-encoded subunits of complexes I and IV gives explanation for toxicity of APP or A*β*, or NFTs in mitochondria. There are additional findings in mitochondria affected by AD such as lower activity of pyruvate dehydrogenase (PDH) and oxoglutarate dehydrogenase (OGDH) [104, 132]. Inhibition of OGDH, the enzyme of tricarboxylic acid cycle, minimizes the NADH pool and electron number needed for ETC and mitochondrial membrane potential (Δ*ψ*_*m*_) to create and maintain proton gradient obligatory for ATP synthesis. There are also lines of evidence for direct inhibitory action of soluble oligomeric A*β* species on ABAD (A*β*-binding alcohol dehydrogenase) and internal membrane cyclophilin D (CypD) resulting in increased mitochondrial membrane permeability (MPTP), potentiated ROS production, synaptic loss, diminished activity of mitochondrial respiration, and finally cell death [129, 132–134]. CypD knockout prevents mitochondrial and neuronal perturbations and improves mitochondrial function in Alzheimer's disease mouse model [128, 135]. It has to be emphasized that oxidative/nitrosative stress affects the fusion and fission process of mitochondria. Fusion, that is, speedup by small GTPases mitofusins (Mfn1/Mfn2), improves efficiency of mitochondrial respiration and ATP production. Mitochondrial dynamics are severely imbalanced in AD cases in favour of fission, through elevated expression of the fission protein DLP1 (dynamin-like protein 1) associated with nitrosative stress stirred up by A*β* [136, 137]. Collectively, these observations indicate that toxic intracellular A*β*_42-43_ oligomers differ in action from extracellular aggregates found in amyloid plaques of AD brains [138] (Figure 3).

## 12. Proteostasis in Mitochondria

Mitochondrial protein turnover grants the well-organized replacement of nonfunctional proteins into operational one. The first task is attained through proteolytic degradation of inner membrane and matrix proteins with local mitochondrial proteases [139] or outer membrane proteins through ubiquitin-proteasome system [140]. The second task is met by intramitochondrial protein synthesis but as almost 1500 different nuclear-encoded proteins have to be imported to complete mitochondrial proteome, a matter of capable import is fundamental for the function of the organelle. In extreme cases of cellular injuries observed in neurodegenerative diseases, damaged mitochondria with extended loss of the electrochemical potential are selectively removed by autophagy known as mitophagy [112]. APP and A*β* accumulation has also something to do with altered mitochondrial dynamics as fission takes advantage of fusion with resultant dysfunction of mitochondria and neurons [137]. AD neurons demonstrated selective mechanisms of proteolytic clearance of oxidatively and nitrosatively modified proteins in mitochondria. Insulin degrading enzyme (IDE) prevents formation of toxic insoluble fibrils from A*β*, as it cleaves A*β* prior to aggregation [141–143]. A novel zinc-metallopeptidase, Presequence Protease (PreP), a member of pitrilysin oligopeptidase family, degrades either intramitochondrially stored A*β*_40_ or A*β*_42_ protein. This protease seems to be highly sensitive to oxidative stress, as disulfide bridge formed between two proximal cysteine residues blocks its catalytic activity [144–149]. Another mitochondrial serine protease HtrA2/Omi occupies intermembrane space where it can cleave APP locked up in protein translocase complex [121]. Even though it is well established that HtrA2/Omi is released to the cytosol to amplify apoptosis through the degradation of antiapoptotic proteins and caspase activation [150–152], it is also implicated in proteolytic deletion of malfolded APP in ER [151]. From knockout studies on mice, it is obvious that HtrA2/Omi plays a significant shielding role as mice deficient in this protease exhibit neurodegenerative phenotype with weight loss and premature death [153]. Taken together, understanding the mechanisms of clearance of the unwanted proteins including APP, A*β*, and tau is vital in an attempt to get rid of them from mitochondria. Some hopes are associated with the application of at present indefinite modulators of proteolytic activity. HtrA2/Omi is, for example, activated by PTEN-induced putative kinase 1 (PINK1) upon phosphorylation at Ser142 residue [154]. Alternatively, accent has to be put on mitophagy of dysfunctional mitochondria and mitochondriogenesis.

## 13. Aging versus AD

The most common neurodegenerative disorder is represented by Alzheimer's disease, characterized by declining memory, reduced cognitive capacity, and progressive dementia, which are often fatal to elderly individuals above 65 years of age. It is ranked as the fourth leading cause of death in modern societies where average life span increased greatly in the last two decades. As 95% of AD cases are diagnosed in older people, one might think that a causal relationship exists between aging and the onset of disease. Certainly, a number of similarities between getting old and being affected with AD could be listed. Historically, the free radical theory of aging by Harman [155] suggested aging as “side effect” of reactive oxygen species formed in mitochondrial respiratory chain. Apparently, free radicals, commonly generated by incomplete reduction of oxygen molecule at complexes I and III of mitochondrial ETC, are capable of damaging DNA, RNA, and proteins. They impair energy storage and lead to operational failure of mitochondria with progressive decline of cell viability. Almost identical conditions accompany AD and are an explicit step in pathogenesis of disease. As mtDNA is deprived of repair mechanisms, ROS-induced DNA strand breaks tend to accumulate with age or AD. Thus, mtDNA is a vulnerable target for ROS, but the reverse, the ROS generation due to the mutated mtDNA, is not convincingly confirmed [156]. Moreover, the evidence that mutated mtDNA accelerates the progress of aging is also questioned based on the results from study carried out on transgenic mice model [157]. Although some authors show inconsistency between the free radical theory and observations, cumulative evaluation of the scientific reports points to antioxidant defense systems as important factors in protection from premature aging [158]. Other mitochondrial components important for their function are hampered with age: adenine nucleotide translocase (ANT), nitric oxide synthase (NOS), and carnitine acyltransferase (CT) [159–161]. Actually, NOS activity is elevated in AD as reported from study performed on cellular model of disease [15]. We could not find any information about CT activity in AD, whereas ANT activity is noticeably inhibited by A*β* or hyperphosphorylated tau and this effect is reversed by mersalyl, a reversible alkylating agent of thiol groups [162]. Mitochondrial dysfunction, observed in transgenic mice models of AD and aging, demonstrates higher activity of genes controlling energy metabolism and apoptosis. Taken together, physiological aging and AD are associated with broad-spectrum dysfunction of mitochondria, but the foundations of mitochondrial decline are dissimilar. More discrepancies between physiological aging and AD were found with respect to ERS and UPR which play a significant role in cellular proteostasis. Dolichol was selected as an aging marker because the progressive increase in dolichol level was observed in aging brain [163]. In contrast, ubiquinone concentration which is also synthesized from geranylgeraniol diminishes with aging whereas cholesterol and dolichyl phosphate concentrations remain unaltered. In AD, decreased levels of dolichol were observed and increased levels of ubiquinone and dolichyl phosphate without any changes in brain cholesterol. AD cannot be regarded as a result of premature aging. The drop in dolichol and augmented dolichyl phosphate concentration points toward disturbed glycosylation in ER of diseased brain, while the increase in ubiquinone suggests efforts to protect the brain from oxidative stress induced by lipid peroxidation [164, 165].

## 14. Targeting ER Stress in AD Therapy

As ERS is a recognized factor in AD, drugs that interfere with ERS would theoretically have great therapeutic potential. There are several compounds grouped in classes that interact directly with components of the ERS (salubrinal, BiP inducer X (BIX), salicylamide analogs, flavonoids, guanabenz, and STF083010), chemical chaperons (4-phenylbutyric acid (PBA), tauroursodeoxycholic acid (TUDCA), and trimethylamine oxide (TMAO)), chemicals that inhibit protein degradation (Eeyarestatin, MG132, and Bortezomib), compounds with antioxidant activity (Edaravone, dibenzoylmethane derivatives, and N-acetyl cysteine (NAC)), and drugs controlling calcium signaling (dantrolene and carbazole derivatives) [166]. They may act by inducing transient translation arrest, upregulation of chaperone proteins, and augmented degradation of ER-associated misfolded proteins. Fundamental approach in the development of new therapy is the selection of appropriate molecular targets. In ER stress signaling, the aim is to alter the expression of ER stress-associated molecules that can rescue cells from the toxic effect of ERS. Recent efforts in establishing new promising drugs against AD are pointing to chemical chaperones such as PBA, TUDCA, or trimethylamine oxide (TMAO). These substances improve protein folding and alleviate native protein conformation [166]. It was shown on mouse models of AD that PBA, TUDCA, and TMAO stop A*β* accumulation and avoid the loss of dendritic spines [167]. Some observations even demonstrated improved memory and cognitive functions [168] associated with improved cell survival [169]. Salubrinal ((2E)-3-phenyl-*N*-[2,2,2-trichloro-1-[[(8-quinolinylamino)thioxomethyl]amino]ethyl]-2-propenamide, Sal) selectively inhibits growth arrest and DNA damage induced gene 34- (GADD34-) phosphatase complex (GADD34 associates with protein phosphatase 1 (PP1)) and promotes* in vitro* dephosphorylation of the alpha subunit of eIF-2*α* and IRE1*α*/ASK1/JNK signaling pathway being protective against ERS even induced by tunicamycin Tm [170]. In a great deal of experiments testing Sal in cultured cells and animal models of AD, this substance increased the viability of neuronal cells and A*β* toxicity [171, 172]. BIX (2-(3,4-dihydroxyphenyl)-2-oxoethyl ester thiocyanic acid) preferentially induced BiP mRNA in an ATF6-dependent manner leading to reduced Tm-induced death of neuronal cells [173]. Also DBM derivative 14–26 (2,2′-dimethoxydibenzoylmethane) was found to be neuroprotective for SH-SY5Y and PC-12 cells by decreasing expression of BiP and CHOP [174]. Dantrolene, a ryanodine receptor antagonist that inhibits abnormal calcium release from the ER, inhibited expression of both phosphorylated PERK and eIF2*α*. It also reduced CHOP expression and attenuated thapsigargin-induced apoptosis in PC-12 cells [175]. Neuroprotective effects similar to dantrolene were observed for ([9-(3-cyanobenzyl)-1,4-dimethylcarbazole]). This substance suppressed increases in intracellular Ca^2+^ in PC-12 cells treated with thapsigargin and reduced levels of BiP and CHOP [176].

Aforementioned compounds were chosen from others as the most potent ER stress inhibitors and persuasively protective to neuronal cells. 18 of 42 different compounds were exploited in* in vivo* and* in vitro* models of central nervous system disorders with, in fact, improved cell or tissue viability [166]. Thus, the brain is the most frequently investigated organ in the context of ERS. From these experiments, it becomes clear that CHOP functions as proapoptotic factor. The roles of other specific ER stress molecules as molecular targets for pharmacological intervention are less clear and vary depending on cell type and context.

There are few underestimated modulations in APP processing that shed more light on current dogma of AD pathogenesis. The modulation of mevalonate pathway and cholesterol synthesis were reported to stimulate nonamyloidogenic pathway of APP processing [177]. Additionally, cholesterol derivative 27-hydroxycholesterol (27-OHC) was shown to induce ER stress which attenuated leptin-dependent viability by activating CHOP in SH-SY5Y neuroblastoma cells [178]. Irrespective of a number of compounds examined in AD, the call for new drugs modulating ER stress with healing effect is still waiting to be revealed.

## Abbreviations

APOE: Apolipoprotein E
APP^s^: Secreted form of APP
CHOP/GADD153: C/EBP homologous protein/growth arrest and DNA damage induced gene 153
Drp1: Dynamin-1-like protein
ERAD: Endoplasmic-reticulum-associated protein degradation
ERO1*α*: ER oxidase 1*α*
ERS: Endoplasmic reticulum stress
FOH: Farnesol
GGOH: Geranylgeraniol
GRP75: Glucose-regulated protein 75
GRP78/BiP: 78 kDa glucose-regulated protein
GSK-3*β*: Synthase kinase 3 beta
IP_3_R: Inositol triphosphate receptor
IRE1*α*: Inositol-requiring enzyme 1 alpha
MAM: Mitochondria-associated membrane
MAP LC3: Microtubule-associated protein light chain 3
Mfn2: GTPase mitofusin 2
MMP: Mitochondrial membrane potential
MPTP: Mitochondrial permeability transition pore
NFT: Neurofibrillary tangles
PACS-2: Phosphofurin acidic cluster sorting protein 2
PDI: Protein disulfide isomerase
PERK: Protein kinase R- (PKR-) like ER kinase
RNS: Reactive nitrogen species
ROS: Reactive oxygen species
RyR: Ryanodine receptor
TOM: Transporter outer membrane
TIM: Transporter inner membrane
UPR: Unfolded protein response
VDAC: Voltage-dependent anion channel
XBP-1: Xbox binding protein 1.
